# Model of the Entrepreneurial Intention of University Students in the Pearl River Delta of China

**DOI:** 10.3389/fpsyg.2019.00916

**Published:** 2019-04-30

**Authors:** Fei Hou, Yu Su, Minru Lu, Mingde Qi

**Affiliations:** ^1^School of Management, Beijing Normal University, Zhuhai, China; ^2^School of Management, Guangdong University of Technology, Guangzhou, China

**Keywords:** entrepreneurial passion, role models, entrepreneurship education, entrepreneurship self-efficacy, entrepreneurial intention

## Abstract

Although the entrepreneurial intention of university students has been studied from different perspectives, the results are still not convergent, and the mechanism and outcomes related to how entrepreneurial intention could be affected by different factors lacking integrated investigation and comparative research. Based on emotional theory, the theory of planned behavior (TPB), and entrepreneurial cognitive theory, as well as the perception of specific situations encountered by university students, this paper attempts to explore entrepreneurial intention from three perspectives, including individual, family and school; and constructs an integrated model that includes entrepreneurial passion, role models, entrepreneurial education, entrepreneurial self-efficacy, and entrepreneurial intention. Based on a survey of university students in the Pearl River Delta of China, this paper attempts to explore the intrinsic mechanism of the development of entrepreneurial intention from these three perspectives. The results show that entrepreneurial passion, role models, and entrepreneurial education could have different effects on entrepreneurial intention; additionally, entrepreneurial self-efficacy plays an important mediating role. The research findings contribute to the literatures regarding the factors influencing entrepreneurial intention, providing empirical evidence to formulate policies to encourage university students’ entrepreneurship practices and help to enhance effectiveness of entrepreneurship education.

## Introduction

In recent years, with the emergence of entrepreneurship activities and incubators, more and more people have focused on the study of entrepreneurship. While university students are often regarded as potential entrepreneurs, entrepreneurial intention is the core variable to predict the entrepreneurial behavior of university students (Krueger et al., 2000).

Regarding the study of entrepreneurial intention, scholars have begun to explore the key factors affecting entrepreneurial intention by using internal factors, such as psychological traits, personal characteristics, and the cognition of entrepreneurs, and have analyzed the development mechanism of entrepreneurial intention (Shapero and Sokol, 1982; Scott, 1991; Kickul and Krueger, 2004); however, these studies ignore the impact of external environmental factors on entrepreneurial intention. Later studies have explored environmental factors affecting entrepreneurs, taking into account both internal and external factors when constructing their entrepreneurial intention model (Ajzen, 1991; Shook, 2003).

However, the current entrepreneurial intention model ignores the impact of specific situations, as entrepreneurs face specific situations that will inevitably affect their internal factors. That is, entrepreneurial intention is not only influenced by carrier factors (individual factors) but also by specific situational factors. Only when carrier factors and situational factors work together can entrepreneurial intention be stimulated (Elfving, 2008).

Therefore, the study of entrepreneurial intention began from a psychological perspective and then gradually combined with internal factors, such as trait theory, resource view and ability view. The study of entrepreneurial intention has now entered a new stage of research from a situational factor viewpoint. The focus of the present study highlights the role that specific situational factors play in the generation and development of entrepreneurial intention. Thus, different entrepreneurs will exhibit different performances in various specific situations; the entrepreneurs’ internal factors and specific situational factors will jointly drive the emergence and development of entrepreneurial intention. However, questions remain: how do these internal and external factors drive entrepreneurial intention, and how do their effects differ? These questions must be explored and interpreted.

According to the relevant literature, the influencing factors of university students’ entrepreneurial intention are divided into two levels: individual factors (micro level) and environmental factors (macro level). The micro-level factors mainly include individual characteristics, psychological characteristics, school experience, entrepreneurial knowledge, and ability, while the macro-level factors mainly include social cultures and norms, policy environment, economic level, personal social network, family background, and entrepreneurial education (Garavan and O’ Cinneide, 1994).

This paper regards university students as potential entrepreneurs. At the level of personality traits, this paper mainly focuses on the emotional level of entrepreneurial passion; at the specific situation level, this paper is concerned with the impact of role models at the family level on university students, and at the school level, this paper chooses entrepreneurship education as the specific situational factor affecting entrepreneurial intention.

At present, entrepreneurial passion based on emotional theory is a research hot spot about the individual factors of entrepreneurial intention and is a key factor affecting entrepreneurial activities (Foo, 2011). Cardon et al. (2013) states that the entrepreneurship process is also the emotional experience process of entrepreneurs. The dimension of entrepreneurial passion includes intense positive feelings and identity centrality, which is the main driving force of entrepreneurial intention. The theory of planned behavior (TPB) is widely used in the field of entrepreneurship. One of the elements constituting the TPB is the subjective norms, which consists of the following two parts: one is the degree to which an individual complies with certain behaviors, and the other is the degree to which a person in an intimate relationship expects certain behaviors of another individual (Azjen, 1991). For university students, from the perspective of specific situations, the subjective normative level is mainly represented by role models. Scott and Twomey (1988) believe that parents have a great influence on an individual’s entrepreneurial intention. Parents, as entrepreneurial models, and their experience will significantly affect entrepreneurial intention. However, current studies have performed limited research on the relationship between role models and entrepreneurial intention, and the results are quite different (Brenner et al., 1991; Gird and Bagraim, 2008; Geldhof et al., 2013). Therefore, based on the subjective normative level of TPB, this paper focuses on the impacts of family role models on entrepreneurial intention.

Moreover, in recent years, with research on entrepreneurial intention focusing on external factors, entrepreneurial education has gradually become a research hotspot. The university’s entrepreneurship atmosphere and the support of entrepreneurship activities will affect the attitude of students toward entrepreneurship. Entrepreneurship education resources and the development of entrepreneurship courses could improve the perceived behavior control variables of university students, thus affecting their entrepreneurial intention.

The entrepreneurship atmosphere created by schools and the support of entrepreneurship activities will affect the attitude of university students toward entrepreneurship and entrepreneurship education resources; the development of entrepreneurship courses could improve entrepreneurial intention. Therefore, the mechanism of entrepreneurship education needs to be further explored; thus, based on the specific situation level, this paper also takes entrepreneurship education as the pre-variable of entrepreneurial intention.

In short, the exploration of influencing factors of entrepreneurial intention based on specific situations has both theoretical and practical implications. From the individual level, family level, and school level, this paper extracts three pre-variables: entrepreneurial passion, role models, and entrepreneurial education. Taking entrepreneurial self-efficacy as a mediating variable, this paper constructs a multi-perspective integration model of entrepreneurial intention. Based on the survey data of university students in the Pearl River Delta region, this paper explores the effects of factors at the individual level, family level, and school level on entrepreneurial intention.

## Literature Review and Research Hypothesis

### Entrepreneurial Passion and Entrepreneurial Intention

Passion was introduced into the field of entrepreneurship by scholars in the 21st century. In the process of entrepreneurship, the difficulties and obstacles entrepreneurs encounter are inevitable, but successful entrepreneurs will persist, which is inseparable from entrepreneurial passion (Zhu et al., 2011). Passion for entrepreneurship is the motivation for individuals to participate in entrepreneurial activities (Bierly et al., 2000) and is a strong and positive emotion that can stimulate individual potential (Baron and Ward, 2004). Passion for entrepreneurship is the source of courage for entrepreneurs to face risks and challenges; this passion presents not only at the emotional level but also at the cognitive level (Cardon et al., 2009).

Entrepreneurial intention is the subjective thinking and mental state of entrepreneurs before they implement entrepreneurial behavior (Krueger et al., 2000). Baron (2008) proposed that a key factor in generating entrepreneurial motivation and entrepreneurial desire is entrepreneurial passion, which can stimulate people’s internal motivation and individual entrepreneurship (Vallerand et al., 2010). Therefore, when entrepreneurial passion is stimulated, emotional expression strengthens. With a high interest in entrepreneurship, the individual’s perception of entrepreneurship will be enhanced, as will the possibility of translating ideas into actions.

Cardon et al. (2013) divides entrepreneurial passion into two dimensions: intense positive feelings and identity centrality. Intense positive feelings are emotional expressions generated when individuals participate in entrepreneurial activities. The higher their interest in entrepreneurial activities is, the more confident they are about uncertainty and challenges, and this affects their entrepreneurial intention. Identity centrality is the identification of an individual to his own entrepreneur. When he accepts this identity, entrepreneurial ideas will also be generated.

Therefore, this study proposes:

H1: There is a positive relationship between entrepreneurial passion and entrepreneurial intention.

### Role Models and Entrepreneurial Intention

Role essentially reflects a kind of social relationship, which is consistent with individual social status and which matches with identity. Roles are aggregates of characteristics of certain groups, which generally refer to the similar goals, attitudes or behaviors of groups.

The role models in the field of entrepreneurship are people with a supportive and encouraging attitude toward entrepreneurship and who also have a certain amount of successful experience. If people interested in learning about entrepreneurship maintain consistency with the goals of their role models, then they are vulnerable to their influence. These people also hope to learn from the experience of role models to achieve a goal. Therefore, role models are worth emulating (Basow and Howe, 1980).

According to the literature, role models are not well-known or authoritative people in the industry but, rather, are family members or friends we meet daily. Role models are closely related to individuals, including parents, relatives, friends, etc. This finding shows that the better and closer the relationships between individuals and their role models are, the more vulnerable the individuals are to their influence (Davidsson, 2004).

Busenitz and Barney (1997) argue that university students’ entrepreneurial intention is influenced by role models. They point out that when parents’ values are conservative and resistant of unstable and risky activities, they strongly discourage their children from participating in entrepreneurship projects. However, when parents are open-minded and take a positive attitude toward entrepreneurship, they will support their children to experience the process of entrepreneurship and participate in entrepreneurship projects. Therefore, individuals could complete entrepreneurship tasks stimulated by role models and thus make decisions. Since role models could positively affect entrepreneurial behavior, their existence is conducive to the formation of entrepreneurial intention in university students.

In addition, university students are vulnerable to the influence from friends who may have similar background resources. When a friend starts a new business and succeeds in it, university students will assume that if they enact the same behavior, then they will achieve the same level of performance, which would affect their entrepreneurial intention. Therefore, role models provide entrepreneurship experience for university students, who are also influenced by role models’ attitudes toward entrepreneurship, so the entrepreneurial intention will improve.

Therefore, this study proposes:

H2: There is a positive relationship between role models and entrepreneurial intention.

### Entrepreneurial Education and Entrepreneurial Intention

Entrepreneurship education could improve the ability of an individual to identify market opportunities and perceived risk (Peterman and Kennedy, 2003). When university students are regarded as potential entrepreneurs, entrepreneurship education is a means of providing entrepreneurship knowledge, cultivating entrepreneurship spirit, and improving entrepreneurship ability and psychological quality. Accordingly, entrepreneurial education has a positive impact on individual entrepreneurship attitude and ability.

Kolvereid and Moen (1997) pointed out that students in an entrepreneurship major have a higher entrepreneurial intention than those from non-entrepreneurship majors, and these students are likely to create new businesses after graduation. According to the literature, it is generally believed that entrepreneurship education has a positive impact on entrepreneurial intention (Rideout and Gray, 2013). In addition, the number of management courses is positively related to university students’ entrepreneurial intention (Chen et al., 1998).

By gaining entrepreneurial knowledge, enhancing entrepreneurial awareness, developing the psychological qualities of entrepreneurship, and improving students’ entrepreneurship ability and their understanding of the entrepreneurial spirit, entrepreneurial education can enhance the entrepreneurial intention of university students.

Therefore, this study proposes:

H3: There is a positive relationship between entrepreneurship education and entrepreneurial intention.

### Entrepreneurial Passion and Entrepreneurial Self-Efficacy

Entrepreneurial self-efficacy is derived from the concept of “self-efficacy,” which was first proposed by Bandura (1977). This involves the individual’s control over the role of entrepreneurship and the strength of belief in accomplishing entrepreneurship tasks (Scherer et al., 1989), as well as the belief that individuals can effectively complete entrepreneurship tasks and achieve the goals of entrepreneurial behavior (Chen et al., 1998).

In addition, entrepreneurial passion could promote individuals to positively evaluate the results of entrepreneurship and to believe that they can achieve success in entrepreneurship. Entrepreneurial passion can influence entrepreneurship cognition, that is, it plays a very positive role in entrepreneurial self-efficacy (Perttula, 2004). Passionate entrepreneurs are more confident in their evaluation of opportunities and in their entrepreneurial abilities. When entrepreneurs’ enthusiasm for entrepreneurship is active, they will constantly seek new markets, more actively explore new products and learn new entrepreneurship knowledge and management knowledge (Cardon et al., 2013).

Cardon et al. (2009) added the concept of identity centrality to the study of entrepreneurial passion, and her research perspective gradually changed from an emotional viewpoint to a cognitive viewpoint. Intense positive feelings and identity centrality construct the dimensions of entrepreneurial passion. Her study showed that identity centrality is an individually perceived entrepreneurial identity from a psychological point of view (Cardon et al., 2013).

In addition, the individual’s enthusiasm and interest in entrepreneurship activities is an intense positive feeling that can improve the individual’s entrepreneurial confidence. Therefore, to stimulate university students’ entrepreneurial passion, that is, to retain entrepreneurs’ positive emotions about entrepreneurial activities and to make entrepreneurs clarify their identity as entrepreneurs, could result in enhancing entrepreneurial self-efficacy.

Therefore, this study proposes:

H4: There is a positive relationship between entrepreneurial passion and entrepreneurial self-efficacy.

### Role Models and Entrepreneurial Self-Efficacy

One of the ways to influence self-efficacy is to set an example of self-efficacy behavior for others. Individuals can enhance their self-efficacy by learning from others’ experience (Bandura and Wood, 1989). By observing the behavior of role models and learning the corresponding skills and methods, individuals can infer how much money, time, and energy they need to invest to achieve a performance similar to that of their role models (Gist and Mitchell, 1992).

Scott and Twomey (1988) found that parents’ entrepreneurial experience can influence an individual’s entrepreneurial cognition. Moreover, as the relationship between parents and university students is extremely close, it can help students to personally feel the process of parents’ entrepreneurship, which will affect their own self-efficacy and confidence. Thus, role models are an important source of university students’ confidence and self-efficacy in entrepreneurial activities.

Therefore, this study proposes:

H5: There is a positive relationship between role models and entrepreneurial self-efficacy.

### Entrepreneurship Education and Entrepreneurial Self-Efficacy

The unstable personal characteristics of self-efficacy will change following tailored education (Hollenbeck and Hall, 2004). At the same time, entrepreneurship is periodic in nature, which also affects the self-efficacy of entrepreneurship. Specifically, when the individual experiences the entrepreneurial process and has a successful entrepreneurial experience, the entrepreneurial self-efficacy will improve; when suffering setbacks, the entrepreneurial self-efficacy will be reduced. Cox et al. (2002) found that students with entrepreneurship education have higher self-efficacy than those without entrepreneurship education.

The quality of entrepreneurs can be cultivated through acquired education. Individual entrepreneurs participating in entrepreneurship training or entrepreneurship courses and activities performed by schools can improve their psychological quality and entrepreneurship ability. That is, when an individual learns entrepreneurship knowledge through entrepreneurship education and experiences the entrepreneurship process through practice, their confidence in their ability to successfully accomplish tasks or surmount challenges will be enhanced, and the evaluation of their own ability will be correspondingly positive.

Therefore, this study proposes:

H6: There is a positive relationship between entrepreneurship education and entrepreneurship self-efficacy.

### Entrepreneurial Self-Efficacy and Entrepreneurial Intention

With the development of cognitive theory, entrepreneurial self-efficacy plays an increasingly significant role in influencing entrepreneurial intention. Entrepreneurial self-efficacy can predict entrepreneurial intention (Franke, 2004) and will affect entrepreneurs’ perception of potential self-confidence and entrepreneurship performance (Chen et al., 1998). Therefore, entrepreneurial self-efficacy is of great importance to entrepreneurial behavior and entrepreneurship activities.

Entrepreneurial self-efficacy is the key variable for an individual to become a real entrepreneur (Chen et al., 1998). It is the main factor affecting entrepreneurs and their behavior. Entrepreneurial self-efficacy is also an intrinsic cognitive trait of an individual. The more self-confident university students are about their own abilities, the stronger their entrepreneurial intention will be. University students participate in entrepreneurial activities and experience the entrepreneurial process; during this time, the sense of achievement they finally acquire will enhance their entrepreneurial self-efficacy. When they believe in successful entrepreneurship, the possibility that they will invest in entrepreneurial projects will be stronger. Thus, entrepreneurial self-efficacy can effectively predict entrepreneurial intention.

Therefore, this study proposes:

H7: There is a positive relationship between entrepreneurial self-efficacy and entrepreneurial intention.

### The Mediating Role of Entrepreneurial Self-Efficacy

Entrepreneurial self-efficacy is a key precedent of individual entrepreneurial intention, and it is the internal factor that decides whether potential entrepreneurs invest in entrepreneurship activities.

Cardon et al. (2009) analyzed the individual’s interest in entrepreneurship projects from an emotional perspective. When they assessed their abilities and market environment, they would transform ideas into practical actions. This shows that entrepreneurial self-efficacy has a mediating effect between entrepreneurial passion and entrepreneurial intention. Individuals with a high passion for entrepreneurship have a positive evaluation of their entrepreneurial ability. Accordingly, they believe in their success, which in turn affects their entrepreneurial intention (Vallerand et al., 2003). Thus, entrepreneurial passion can enhance an individual’s confidence and willingness to start a business.

Therefore, this study proposes:

H8: Entrepreneurial self-efficacy plays a mediating role between entrepreneurial passion and entrepreneurial intention.

Bandura (1977) believed that individual self-efficacy would improve when a model with similar conditions succeeded through hard effort. When an individual observes the successful experience of others, he will infer the ability he needs to achieve the same level of achievement, which will have an impact on his ability evaluation. Accordingly, role models are helpful in improving an individual’s entrepreneurial self-efficacy, promoting an individual’s positive performance and increasing the possibility of entrepreneurship (Gibson, 2004). Thus, role models help to enhance individual entrepreneurial confidence and make individuals more willing to participate in entrepreneurship activities.

Therefore, this study proposes:

H9: Entrepreneurial self-efficacy plays a mediating role between role models and entrepreneurial intention.

Entrepreneurship education can enhance an individual’s entrepreneurial confidence and influence their entrepreneurial intention (Chen et al., 1998). Individuals acquire entrepreneurial knowledge and skills through entrepreneurship education, which will increase their entrepreneurial awareness and their entrepreneurial understanding (Wilson et al., 2007). This knowledge will also enhance their entrepreneurial self-efficacy and affect their entrepreneurial intention. That is, entrepreneurship education can cultivate individual entrepreneurship ability and psychological quality and affect entrepreneurial intention by enhancing entrepreneurial self-efficacy.

Therefore, this study proposes:

H10: Entrepreneurial self-efficacy plays a mediating role between entrepreneurship education and entrepreneurial intention.

In short, specific situational factors affect entrepreneurial intention, but external factors cannot directly affect entrepreneurial intention but, rather, indirectly affect entrepreneurial intention through personal cognition (Krueger et al., 2000). Therefore, this paper proposes that entrepreneurial passion, role models and entrepreneurship education can stimulate entrepreneurship self-efficacy. According to entrepreneurial cognitive theory, entrepreneurial self-efficacy has been indicated to be the key factor in effectively predicting entrepreneurial intention (Boyd and Vozikis, 1997).

**FIGURE 1:**
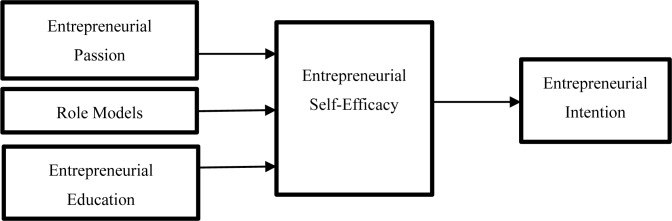
Proposed theoretical model.

Based on the above analysis, the proposed concept framework constructed in this paper is shown in Figure 1.

## Materials and Methods

### Procedure

Currently, universities in China may be seen as an intensive source of knowledge support and entrepreneurial opportunities. Universities in China try to provide several university policies and adequate environments where the university community can explore, evaluate and exploit knowledge that could be transformed into new ventures. Therefore, it is for this reason that it makes sense for intentions to be studied in this specific knowledge context (students at university).

This study uses a quantitative approach rather than a qualitative approach because it attempts to seek empirical support for hypotheses developed from previous literature. The data collected and the results from the study will predict the relationship between the variables hypothesized.

The data used in this study were collected using a primary source (questionnaire). The questionnaire used in this research was adapted from earlier studies. Questionnaires were distributed to university students in the Pearl River Delta of China, mainly in the cities of Guangzhou, Shenzhen, and Zhuhai. The university students answering the questionnaire must be those who have taken an entrepreneurship course.

The questionnaire consists of three parts. Part A seeks information on the respondent’s socio-demographic data. Part B obtains information about the factors that influence the respondent’s intention to be an entrepreneur. Part C solicits the respondent’s intention to be an entrepreneur. The questionnaires were back-translated into the language of origin to assure no loss of meaning. The questionnaires were administered during class sessions, yielding a response rate of 100%. Using Likert scales and demographic variables, we measured students’ entrepreneurial intentions as well as their perceived barriers to business start-up.

All surveys in our study were anonymous and did not include any individual identification elements and did not violate the privacy of the research participants. Moreover, respondents were assured of the anonymous nature of the data collection effort in advance. All respondents were informed that participation was voluntary, and that confidentiality was ensured.

The data were checked for reliability, validity, normality, and multicollinearity. Hierarchical multiple regression analysis and independent *t*-tests were used to analyze the data.

### Sample Selection and Data Collection

The Pearl River Delta region of Guangdong province is one of the most developed regions in China and is a leading region in the process of China’s reform and opening. During a period of more than 40 years spent contributing to this process, the Pearl River Delta region attracted a large number of entrepreneurs from all over the country and, even, all over the world who have started businesses. These entrepreneurs and their entrepreneurial activities, attracted by the reform and opening, have promoted the rapid economic and social development of the Pearl River Delta.

Therefore, the Pearl River Delta region has a strong entrepreneurial cultural atmosphere and a large number of entrepreneurial practices, which will directly or indirectly exert a subtle influence on university students in this region. At the same time, universities in the Pearl River Delta region pay more attention to entrepreneurship education. There are abundant entrepreneurship-related education courses and practical activities in universities, and students’ entrepreneurial intention is relatively higher here than in other regions in China. Therefore, a study of the entrepreneurship education of university students in the Pearl River Delta has a good typicality and is an ideal sample area for entrepreneurship education and intention research.

For this reason, our research was conducted in universities in the southern cities of the Pearl River Delta of China. Through paper-based and online-based questionnaires, students mainly in Guangzhou, Shenzhen, and Zhuhai were surveyed.

Respondents were told to fill in the questionnaire anonymously and that the responses were only for academic research; these statements were made to prevent the influence of uncertainties on the questionnaire and to ensure the validity of the questionnaire. One hundred eighty-three paper-based questionnaires were distributed on the spot, and 160 were collected. Additionally, 617 questionnaires were collected from the network platform; finally, 800 questionnaires were sent out, and 727 valid questionnaires were collected.

The results of the sample composition are shown in Table 1. The data shows that Guangzhou, Shenzhen, and Zhuhai are the main locations and are typical representative areas of the Pearl River Delta that are active in entrepreneurship activities. Thus, university students in these three locations have more contact with entrepreneurship activities and are more vulnerable to the impact of relevant entrepreneurship information.

**Table 1:** Demographic characteristics.

		*N*	Percentage %
Gender	Male	226	31.1%
	Female	501	68.9%
Grade	Year 1	37	5.1%
	Year 2	134	18.4%
	Year 3	292	40.2%
	Year 4	243	33.4%
	Graduates	21	2.9%
Location	Guangzhou	347	47.7%
	Shenzhen	198	27.2%
	Zhuhai	182	25.1%
Major	Technology	111	15.3%
	Business	524	72.1%
	Art	79	10.9%
	Other	13	1.7%
Entrepreneurial experiences	Yes	131	18.0%
	No	596	82.0%

### Measurement

All measures applied in this research were matured measurement scales which were deemed suitable as they seek to capture constructs that are defined. These measures were developed originally in English and were translated into Chinese and back-translated into English by bilingual experts. Aiming for equivalence and agreement, the back-translated English version was compared with the original English version (Brislin et al., 1973).

### Entrepreneurial Passion

Entrepreneurial passion was measured using a 5-point Likert-type scale with 13-item, which were developed by Cardon et al. (2013) with intense positive feelings and identity centrality as dimensions. A sample measuring item for EP is, “It’s exciting to find solutions to unmet market demands and commercialize them.” (1 = strongly disagree, 5 = strongly agree).

### Role Models

The role models were measured by a scale developed by Obschonka et al. (2011). According to the scale, respondents were asked whether their parents, relatives, or close friends of the family were self-employed by using two items. The first item (targeting a parent’s self-employed status) was recoded into 0 = no and 2 = yes and then summed with the second item (targeting number of self-employed relatives and close friends of the family on a three-point scale; 0 = nobody, 1 = some, 2 = many) so as to calculate the final variable. Consequently, this variable ranged from 0 to 4. Thus, the coding procedure focused on the proximity of parents as role models and the importance of a portfolio of role models simultaneously (Gibson, 2004).

### Entrepreneurship Education

Entrepreneurial education was measured using a 5-point Likert-type scale with 10-item, which were developed by Franke (2004) to analyze the students’ perceived entrepreneurship education environment. The scale encompasses three dimensions as followings: the atmosphere of entrepreneurship education, psychological quality education and curriculum and activity development. A sample measuring item for EE is, “The creative atmosphere in the university inspires us to develop ideas for new businesses.” (1 = strongly disagree, 5 = strongly agree).

### Entrepreneurial Self-Efficacy

Entrepreneurial self-efficacy was measured using a 5-point Likert-type scale with 6-items, which were developed by Chen et al. (1998) and DeNoble et al. (1999). The items on the self-assessment scale broadly represent the extent to which respondents believe in their competencies to cope with uncertainty, change and risk in related to business/entrepreneurial success (Forbes, 2005). A sample measuring item for ESE is, “I believe that I can constantly discover new markets and provide new products or services to meet customer needs.” (1 = strongly disagree, 5 = strongly agree).

### Entrepreneurial Intention

Entrepreneurial intention has been measured through a 5-point Likert-type scale with 5 items, to evaluate the degree to which the research participants have an intention to start a business in the future. The scale to capture EI constructs were adopted from Liñán and Chen (2009); Kuckertz and Wagner (2010). A sample measuring item for EI is, “I am determined to create a firm in the future.” (1 = strongly disagree, 5 = strongly agree).

### Control Variable

With respect to existing studies on the factors affecting EI, this study takes gender (GEN) and entrepreneurial experiences (EEX) as control variables. Individuals with masculine characteristics are associated with potential entrepreneurs and then entrepreneurship is considered as a masculine field according to the researches at the intersection of GEN and entrepreneurship (Ahl, 2006; Gupta et al., 2009; Hu et al., 2018; Wu et al., 2019). Students with more entrepreneurial experience or work experience might be more inclined to consider starting a business (Kautonen et al., 2015).

## Results

### Common Method Variance

According to Fuller et al. (2016), one of the methodological sources of measurement error is common method variance (CMV), which has the potential to harm the reliability and validity of underlying constructs and their postulated correlations in the research model.

With the aim of assessing CMV, both procedural and statistical techniques were applied to lessen its potential effects. For procedural techniques, this study designs an effective questionnaire in which the following techniques were used: (1) research participants were informed about the anonymity and confidentiality of the research survey; (2) honest answers are preferred for the research survey; (3) no right or wrong answer exists in the research survey; (4) ambiguous concepts were avoided; (5) question items were concise; and (6) some reverse-scored question items were designed. Method biases would be expected to lessen by using these procedural measures (Lindell and Whitney, 2001; Podsakoff et al., 2003).

In terms of statistical controls, according to Harman’s one-factor test method, the test results indicate that when the variables in the model were simultaneously loaded into the exploratory factor analysis (EFA), all items of the variables were automatically aggregated into six factors whose eigenvalues are over one. The first emerging unrotated factor, with an eigenvalue of 13.48, cumulatively accounted for 38.52% of the overall variance, which is much lower than the 50% threshold recommended by Podsakoff et al. (2003).

In short, both the procedural and statistical controls above support the conclusion that CMV is not strong enough to bias this study.

### Reliability and Validity

The adequacy of the measurement scales is evaluated based on scale reliability and validity by examining the criteria of reliability, convergent validity, and discriminant validity. Since the role models scale has only two items, the calculation method for the scale of role models differs from general scales, which measure the distance from the relatives around the entrepreneurial activities. The results of the reliability and validity tests are shown in Table 2. The test results show that all scales in the study have acceptable internal consistency and overall validity.

**Table 2:** Measurement items (reliability, convergent validity, discriminant validity).

Construct (source)	Items	Factor loading	SMC	Cronbach’ alpha	CR	AVE
**Entrepreneurial**	EP1	0.842	0.709	0.943	0.959	0.644
**passion**	EP2	0.837	0.701			
(Cardon et al., 2013)	EP3	0.829	0.687			
	EP4	0.814	0.663			
	EP5	0.795	0.632			
	EP6	0.769	0.591			
	EP7	0.724	0.524			
	EP8	0.842	0.709			
	EP9	0.834	0.696			
	EP10	0.831	0.691			
	EP11	0.798	0.637			
	EP12	0.793	0.629			
	EP13	0.714	0.510			
**Entrepreneurial**	EE1	0.822	0.676	0.931	0.941	0.617
**education**	EE2	0.808	0.653			
(Franke, 2004)	EE3	0.800	0.640			
	EE4	0.797	0.635			
	EE5	0.794	0.630			
	EE6	0.784	0.615			
	EE7	0.771	0.594			
	EE8	0.770	0.593			
	EE9	0.765	0.585			
	EE10	0.739	0.546			
**Entrepreneurial**	ESE1	0.852	0.726	0.912	0.933	0.700
**self-efficacy**	ESE2	0.850	0.723			
(Chen et al., 1998;	ESE3	0.837	0.701			
DeNoble et al., 1999)	ESE4	0.834	0.696			
	ESE5	0.826	0.682			
	ESE6	0.819	0.671			
**Entrepreneurial intention**	EI1	0.889	0.790	0.876	0.917	0.734
(Liñán and Chen, 2009;	EI2	0.887	0.787			
Kuckertz and Wagner, 2010)	EI3	0.825	0.681			
	EI4	0.824	0.679			

*SMC, Square multiple correlation, CR, Composite reliability, AVE, Average variance extracted.*

In terms of reliability, this study employed the criteria of Cronbach’s alpha and composite reliability (CR). The values of Cronbach’s alpha of the constructs in the models range from 0.876 to 0.943; the coefficients of CR range from 0.917 to 0.959. Based on this assessment, Cronbach’s alpha and CR coefficients are all above the recommended cut-off of 0.7. Based on this assessment, the model’s constructs exhibit a high internal consistency (Fornell and Larcker, 1981).

In terms of convergent validity, this study applied average variance extracted (AVE) criteria. The results show that the standardized factor loadings of measurement items of scales are significantly over the required minimum of 0.7. In addition, square multiple correlations (SMC) are over the required threshold level of 0.5, indicating the model’s constructs have acceptable item reliability. Values of AVE of constructs in the model range from 0.617 to 0.734, which are all over the recommended threshold level of 0.5. Therefore, the amount of the construct’s variance explained by measurement items of models’ variables is greater than the amount of variance produced by measurement error (Fornell and Larcker, 1981). Based on this assessment, the model’s constructs exhibit a high convergent validity.

In terms of discriminant validity, the square roots of the AVE compare with the inter-correlations of the model’s constructs (Fornell and Larcker, 1981). The results show that the correlation coefficients of each pair of constructs are all lower than the square root of the AVE (see Table 3, shown on the diagonal with bold values in brackets), which indicates that the model’s constructs have discriminant validity.

**Table 3:** Correlations and discriminant validity by Fornell–Larcker criterion.

Construct	Mean	*SD*	1	2	3	4	5
1. EP	3.872	0.678	(**0.802**)				
2. EE	3.587	0.671	0.421^∗∗∗^	(**0.785**)			
3. RM	1.130	1.090	0.117^∗∗∗^	0.036	–		
4. ESE	3.517	0.657	0.606^∗∗∗^	0.523^∗∗∗^	0.095^∗^	(**0.837**)	
5. EI	3.139	0.814	0.535^∗∗∗^	0.317^∗∗∗^	0.124^∗∗∗^	0.591^∗∗∗^	(**0.857**)

*^∗∗∗^Significant at the 0.001 level; ^∗∗^significant at the 0.01 level; ^∗^significant at the 0.05 level. Diagonal elements (bold) in brackets are the square roots of AVEs. Below the diagonal elements are the correlations between the constructs. Variable definition: EP, Entrepreneurial passion; RM, Role models; EE, Entrepreneurial education; ESE, Entrepreneurial self-efficacy; EI, Entrepreneurial intention.*

### Correlation Analysis

The descriptive statistics and the pairwise correlation coefficient of all measured variables in the study are presented in Table 3. Observation indicates that there is little multicollinearity problem, as the intercorrelations among the model’s constructs are below the threshold value of 0.8 (Gujarati, 2003). The results show that there are significant positive correlations among the model’s variables.

**FIGURE 2:**
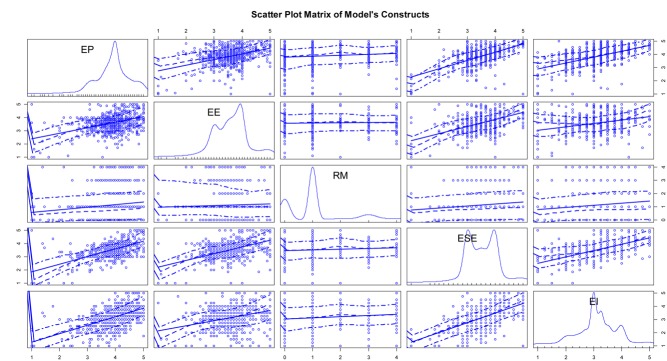
Scatter plot matrix of the model’s constructs. Variable definition: EP, Entrepreneurial passion; RM, Role models; EE, Entrepreneurial education; ESE, Entrepreneurial self-efficacy; EI, Entrepreneurial intention.

To further interpret the correlation of variables in the proposed model, the scatter plot matrix is provided in Figure 2. The graph includes kernel density and rug plots in the principal diagonal and linear and loess fit lines of the model’s constructs. The results show that all independent variables were positively correlated to the dependent variable (EI), and all independent variables were also closely related to the mediator variable (ESE).

On the basis of correlation analysis, hierarchical regression analysis is employed to examine the causal relationship of the model’s constructs.

### Hierarchical Regression Analysis

This section constructs 6 regression models to verify research hypotheses through multiple regression analysis, as shown in Table 4.

**Table 4:** Summary of hierarchical regression analysis.

	ESE		EI			
	Model 1	Model 2	Model 3	Model 4	Model 5	Model 6
GEN	0.166^***^	0.112^***^	0.252^***^	0.162^***^	0.207^***^	0.165^***^
EEX	0.156^***^	0.098^***^	0.202^***^	0.117^***^	0.153^***^	0.117^***^
EP		0.466^***^			0.447^***^	0.279^***^
RM		0.028			0.063^*^	0.050
EE		0.326^***^			0.108^**^	-0.013
ESE				0.543^***^		0.376^***^
*R*^2^	0.054	0.477	0.111	0.390	0.369	0.443
Δ*R*^2^	0.054	0.423	0.111	0.279	0.258	0.074
F-statistics	13.810^***^	109.467^***^	30.151^***^	115.365^***^	70.099^***^	81.574^***^
Durbin–Watson statistic	1.900	1.986	1.932	1.914	1.880	1.874

*N = 727. ^∗^p < 0.05; ^∗∗^p < 0.01; ^∗∗∗^p < 0.001. Variable definition: GEN, Gender; EEX, Entrepreneurial experience; EP, Entrepreneurial passion; RM, Role models; EE, Entrepreneurial education; ESE, Entrepreneurial self-efficacy; EI, Entrepreneurial intention.*

Models 1 and 3 only include the control variables as GEN and EEX. The control variables GEN and EEX both appear to be significantly related to ESE (β = 0.166, *p* < 0.001; β = 0.156, *p* < 0.001), which could explain 5.4% of the total variance in Model 1 (*R*^2^ = 0.054, *p* < 0.001). In addition, the control variables GEN and EEX both appear to be significantly related to EI (β = 0.252, *p* < 0.001; β = 0.202, *p* < 0.001), which could explain 11.1% of the total variance in Model 3 (*R*^2^ = 0.111, *p* < 0.001).

Model 5 is used to verify the main effect of the proposed model, the relationship among EP, RM, EE, and EI. The results indicate that EP, RM, and EE all have a significantly positive relation with EI (β = 0.447, *p* < 0.001; β = 0.063, *p* < 0.05; β = 0.108, *p* < 0.01), which could explain 36.9% of the total variance (*R*^2^ = 0.369, *p* < 0.001); thus, H1, H2, and H3 are confirmed. The implications of the regression results are that EP, RM, and EE could be effective predictors of EI after controlling for the effects of control variables. In addition, the results also show that EP has the greatest predictive effect on EI, followed by EE, and then RM.

Model 2 examines the relationship among EP, RM, EE, and ESE. The regression coefficients are 0.466 (*p* < 0.001), 0.028 and 0.326 (*p* < 0.001), respectively, which could explain 47.7% of the total variance in Model 1 (*R*^2^ = 0.477, *p* < 0.001). The regression results show that EP, EE had a significant positive effect on ESE; thus, H4 and H6 are confirmed. As the regression coefficient of RM is not significant, H5 is not supported.

Model 4 examines the relationship between ESE and EI. The regression coefficients are 0.543 (*p* < 0.001), which could explain 39.0% of the total variance in Model 1 (*R*^2^ = 0.390, *p* < 0.001). The regression results show that ESE had a significantly positive effect on EI; thus, H7 is confirmed.

The mediating effects of ESE on the relationship among EP, RM, EE, and EI have been tested through a second hierarchical regression model analysis; that is, Models 4, 5, and 6 jointly examine the indirect effects of ESE. According to Models 5 and 6, in terms of the EP-EI link, the results show that EP has a significantly positive effect but that the regression coefficient decreases from 0.447 (*p* < 0.001) to 0.279 (*p* < 0.001). In addition, the regression coefficient of ESE was 0.376 (*p* < 0.001), indicating that ESE has a partial mediating effect on the EP-EI link; thus, H8 is verified. For the RM-EI link, since RM has no significantly positive effect on the ESE, ESE has no mediating effect on the RM-EI link; thus, H9 is not supported. For the EE-EI link, the results show that EE has a significantly positive effect (β = 0.108, *p* < 0.01) in Model 5. However, when ESE is added to Model 6, the regression coefficient of EE is not significant, indicating that ESE has a complete mediating effect on the EE-EI link; thus, H10 is verified.

In sum, the first part of the hierarchy regression (Models 1 and 3) tested the effects of control variables. The second part of the hierarchy regression (Model 5) examined the main effect of the independent variables (EP, RM, and EE). The third part of the hierarchy regression (Models 4, 5, and 6) inspected the mediating effects of ESE. The results show that ESE has a partial mediating effect on EP and EI and has a complete mediating effect on EE and EI.

### Indirect Effects Analysis

Traditionally, a four-step hierarchy regression approach is used to examine the mediation effects (Baron and Kenny, 1986). With the prerequisite that the direct path (c path) between the predictor and outcome variable is significant, then the hierarchy regression analysis is to only test whether the independent variable was significant related to mediating variable (a path) and whether the mediator was significant related to dependent variable (b path). In the final step of hierarchy regression, loss or decrease of the direct path between the independent variables and dependent variables was to demonstrate partial or full mediation. However, the requirement of a significant c path makes this hierarchy regression approach suboptimal, because the indirect effects of mediator can occur without the direct effect (Hayes, 2009).

To further test the mediating effects of ESE (Entrepreneurial Self-Efficacy), this study applies a mediating model (Preacher and Hayes, 2008) through the PROCESS SPSS computational tool (Hayes, 2012). One of the advantages of PROCESS is that it provides an actual test of the mediation including bootstrapping to quantify the stability of the indirect effect. With aim to boycott violation of normal distribution assumption, it is recommended to apply a non-parametric bootstrapping approach to evaluate the indirect effects, given the limited sample size (Preacher and Hayes, 2008). A confidence interval for the indirect effect is provided by the bootstrapping method. If the confidence interval (including lower limit and upper limit) of 95% CI (Confidence Interval) do not contain zero, indicating that indirect effects are significant (Preacher and Hayes, 2008).

As for the EP-EI and EE-EI links, according to the normal theory test for indirect effects, the Sobel *Z*-value is 8.943 (*p* < 0.001) and 7.695 (*p* < 0.001), respectively, thus supporting H8 and H10. In addition, bootstrapping results show that significance can be seen in that the 95% CI for the bootstrapping sample does not overlap the zero point (see Table 5, CI: 0.051 to 0.099, and 0.049 to 0.091), indicating that ESE does reliably mediate the relationship between EP and EI, and the relationship between EE and EI. Thus, H8 and H10 are further verified. For the RM-EI link, the Sobel *Z*-test is not significant, and the bootstrapping results show the 95% CI for the coefficient contain zero (see Table 5, CI: -0.038 to 0.121), revealing that ESE does not mediate the relationship between RM and EI; thus, H9 is not supported.

**Table 5:** Bootstrap coefficients, standard errors, and confidence intervals for mediation test.

Dependent variables	Independent variables						

**Direct effect**		**Effect**	**SE**	***t***	***p***	**LLCI**	**ULCI**
EI	EP	0.103	0.014	7.621	0.000	0.077	0.130
	RM	0.152	0.087	1.754	0.080	-0.018	0.322
	EE	-0.014	0.017	-0.815	0.415	-0.046	0.019

**Indirect effect**		**Effect**	**BootSE**	**Sobel *Z***	***p***	**95% CI for Bootstrap**	**95% CI for Bootstrap**
						
						**Lower**	**Upper**

EI	EP	0.074	0.012	8.943	0.000	0.051	0.099
	RM	0.037	0.040	1.019	0.308	-0.038	0.121
	EE	0.068	0.011	7.695	0.000	0.049	0.091
**Contrasts**		

Indirect effect comparison		**Estimate**	**SE**	***Z***	***p***	**95% CI for Bootstrap**	**95% CI for Bootstrap**
						
						**Lower**	**Upper**

IE (EP) vs. IE (EE)		0.006	0.016	0.377	0.706	-0.025	0.037

*Five thousand bootstrap samples with 95% CI. Variable definition: EP, Entrepreneurial passion; RM, Role models; EE, Entrepreneurial education; ESE, Entrepreneurial self-efficacy; EI, Entrepreneurial intention; IE, Indirect effect.*

In addition, due to the significance of the direct effect of EP on EI, ESE (Entrepreneurial Self-Efficacy) partially mediates the relationship between EP (Entrepreneurial Passion) and EI (Entrepreneurial Intention). In addition, owing to the non-significance of the direct effect of EE on EI, ESE fully mediates the relationship between EE (Entrepreneurial Education) and EI (Entrepreneurial Intention).

Therefore, ESE has a partially mediating role between EP and EI and has a fully mediating role between EE and EI. It is of interest to explore whether there exist significant differences between two indirect effects. By conducting a pairwise contrast of two indirect effects, the bootstrapping results show that the 95% CI for the contrast overlapped the zero point (see Table 5, CI: -0.025 to 0.037), indicating that a magnitude of effects cannot be distinguished to compare with the two indirect effects.

### Analysis of Regional Differences

To further explore the research variables in the proposed model, this paper applies ANOVA to investigate the differences among research variables in the survey regions, as presented in Table 6.

**Table 6:** ANOVA test.

		Sum of squares	df	Mean square	*F*	*p*-value
EP	Between groups	958.165	2	479.082	6.261	0.002
	Within groups	55397.942	724	76.516		
	Total	56356.10729	726			
EE	Between groups	553.936	2	276.968	6.242	0.002
	Within groups	32122.650	724	44.368		
	Total	32676.586	726			
RM	Between groups	40.159	2	20.079	17.688	0.000
	Within groups	821.899	724	1.135		
	Total	862.058	726			
ESE	Between groups	232.119	2	116.060	7.614	0.001
	Within groups	11035.550	724	15.242		
	Total	11267.670	726			
EI	Between groups	103.390	2	51.695	4.935	0.007
	Within groups	7583.991	724	10.475		
	Total	7687.381	726			

*N = 727. ^∗^p < 0.05; ^∗∗^p < 0.01; ^∗∗∗^p < 0.001. Variable definition: EP, Entrepreneurial passion; RM, Role models; EE, Entrepreneurial education; ESE, Entrepreneurial self-efficacy; EI, Entrepreneurial intention.*

The results show that there are significant differences among Entrepreneurial Passion (*F* = 6.261, *p* < 0.01), Entrepreneurial Education (*F* = 6.242, *p* < 0.01), Role Models (*F* = 17.688, *p* < 0.001), entrepreneurial self-efficacy (*F* = 7.614, *p* < 0.01), and entrepreneurial intention (*F* = 4.935, *p* < 0.01). The results of the ANOVA test show that there are significant differences among the research variables among university students in Guangzhou, Shenzhen, and Zhuhai.

**FIGURE 3:**
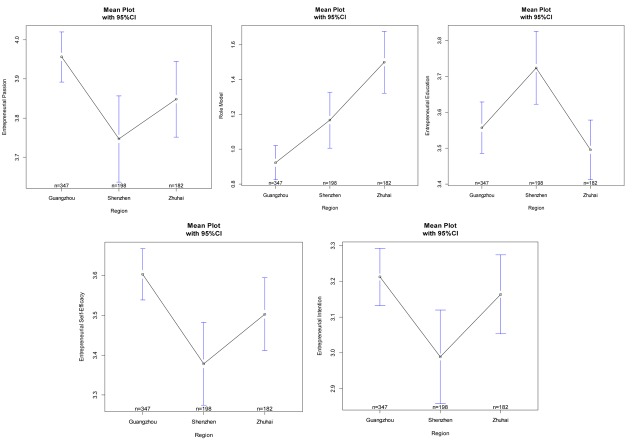
Mean comparison of EP, RM, EE, ESE, EI in terms of regions (Guangzhou, Shenzhen, Zhuhai). Variable definition: EP, Entrepreneurial passion; RM, Role models; EE, Entrepreneurial education; ESE, Entrepreneurial self-efficacy; EI, Entrepreneurial intention.

According to Figure 3, university students in Guangzhou, in comparison with those in Shenzhen and Zhuhai, have a high level of EI, a low level of RM, and a medium level of EE among three predictors of the proposed model. With regard to the mediator of the proposed model, they have a higher level of ESE and have the expected high level of EI.

Regarding university students in Zhuhai, among three predictors of the proposed model, they have a medium level of EP, a high level of RM, and a low level of EE. In addition, they have both medium levels of ESE and EI.

Regarding university students in Shenzhen, among three predictors of the proposed model, they have a low level of EP, a medium level of RM, and a high level of EE. However, they have both unexpected low levels of ESE and EI.

## Discussion

Based on emotional theory, planned behavior theory and cognitive theory, this paper constructs a model of entrepreneurial intention from three perspectives: individual, family and school. Based on a survey of university students in the Pearl River Delta in China, the results show that entrepreneurial passion could both directly and indirectly affect entrepreneurial intention through entrepreneurial self-efficacy and that entrepreneurship education could indirectly affect entrepreneurial intention through entrepreneurial self-efficacy. However, role models have no significantly positive effect on the entrepreneurial self-efficacy, thus entrepreneurial self-efficacy has no mediating effect on the RM-EI link.

Findings from this study are as follows. There is a positive effect between entrepreneurial passion and entrepreneurial intention, which shows that entrepreneurial passion can effectively stimulate the entrepreneurial intention of students. Entrepreneurship education can positively affect entrepreneurial intention. When students receive more entrepreneurship education, their entrepreneurial intention is higher. This shows that entrepreneurship education in universities can cultivate students’ entrepreneurial spirit, create a strong entrepreneurial atmosphere to stimulate students’ entrepreneurial interest and enthusiasm, and positively affect their entrepreneurial behavior. Thus, to stimulate their intention in future entrepreneurial activities, it can be inferred that their individual characteristics (such as entrepreneurial passion) and entrepreneurial education training and program could play a dominant role.

In addition, the findings of this study show that role models have a non-significant impact on entrepreneurial self-efficacy, which indicates that the entrepreneurial intentions of university students are not indirectly affected by family members with entrepreneurship experience through entrepreneurial self-efficacy. For university students, their parents and relatives who have successful experiences have a limited influence on their entrepreneurial intention development. These results may be explained in the following ways. First, role models may transmit negative experiences or signals to respondents, such as role models’ long-time work experiences, discussing business at home, or business failure experiences, which would be expected to discourage the entrepreneurial self-efficacy and entrepreneurial intention of respondents. Second, role models may not offer respondents direct involvement in entrepreneurial activities, but the respondents could gain insights into the nature of entrepreneurship through such direct involvement. Third, direct involvement by the respondents could also provide an opportunity for role models and respondents to enjoy active interaction rather than passive interaction by observation. Thus, such active interaction could in turn help to influence the entrepreneurial self-efficacy and entrepreneurial intention of respondents (Auken, 2006).

Furthermore, the findings show that entrepreneurial self-efficacy has a mediating role between entrepreneurial passion and entrepreneurial intention. This result shows that when individuals are interested in entrepreneurship activities and maintain a stable mood, they will pay attention to entrepreneurship activities, believe in their ability to control resources and complete their entrepreneurship tasks. When faced with entrepreneurship information, they will accept their identity as entrepreneurs and decision makers, improve their sense of self-efficacy, and, thus, enhance their entrepreneurial intention.

Regarding the effects of entrepreneurial education on entrepreneurial intention, entrepreneurial self-efficacy plays a complete mediating role between them. Specifically, entrepreneurial education can indirectly improve students’ entrepreneurial intention through entrepreneurial self-efficacy. Thus, students’ intention to engage in entrepreneurial activities will be influenced by entrepreneurial education contingent on the effectiveness of entrepreneurial training and programs to enhance their entrepreneurial self-efficacy.

As for the regional analysis, universities in Shenzhen were included in the research survey. These institutions attach great importance to entrepreneurship education. The entrepreneurship education system utilized in the universities in Shenzhen is more matured and sophisticated than those in Guangzhou and Zhuhai. Therefore, according to the ANOVA test, the average perceived level of entrepreneurship education of university students in Shenzhen is the highest, followed by Guangzhou and, lastly, Zhuhai. However, the entrepreneurial intention, passion and self-efficacy of university students in Shenzhen are lower. Thus, entrepreneurial education has not impacted entrepreneurial intention. The reasons for this difference could be explored from the aspects of regional entrepreneurship atmosphere and regional economic development, supporting policies, geographical location, and so on.

In this case, this phenomenon could be interpreted by using the proposed intention-based model. Based on the proposed model, we find that entrepreneurial education has significant indirect impacts on entrepreneurial intention. In particular, entrepreneurial self-efficacy plays a complete mediating role in the path of entrepreneurial education to intention. According to the ANOVA test, the mean of entrepreneurial self-efficacy of university students in Shenzhen is the lowest among the regions. That is, although the level of entrepreneurial education is highest in Shenzhen, it fails to have a direct effect on entrepreneurial intention.

Based on the proposed model, entrepreneurial education could indirectly affect entrepreneurial intention through the mediator of entrepreneurial self-efficacy. Therefore, it can be inferred that the entrepreneurship education system should not only provide a series of entrepreneurship courses and practical projects for improving students’ perception of entrepreneurial activities but should also provide training for a variety of special skills and techniques to enhance student self-efficacy when undertaking entrepreneurial activities.

## Conclusion

### Research Implications

The purpose of the study is to explore the antecedents to predict entrepreneurial intention of university students mediated by entrepreneurial self-efficacy. The findings of this study have several theoretical implications for entrepreneurial research and education.

First, the development of the intention to be an entrepreneur is a complex process (Krueger and Kickul, 2006), which is both affected by internal and external factors. Most previous studies have focused on the impact of environmental dynamics on the relationship between antecedents and entrepreneurial intention (Baron, 2008). However, it is difficult to fully reveal the relationship only from external perspectives. In terms of internal perspectives, other studies on the antecedent variables of entrepreneurial intention mainly focus on motivation, subjective norms, and entrepreneurial attitudes (Ajzen, 1991; Sheldon et al., 2006). Therefore, this study further enriches the research on the antecedents of entrepreneurial intention of university students by constructing a research model from the combined internal and external perspectives. The findings of this study indicate that prediction of the entrepreneurial intention of university students is contingent on the specific contexts involved. Thus, in terms of specific contexts, this study proposes the intention-based path model in which university students’ entrepreneurial intentions take shape, including the EP-EI (individual level), RM-EI (family level), EE-EI (school level) path links.

Second, from the cognitive and emotional perspectives, this study explores the relationship between entrepreneurial passion and entrepreneurial intention. In particular, entrepreneurial self-efficacy considered as important cognitive abilities is taken as a mediator in the research model. The findings reveal that entrepreneurial passion could directly enhance entrepreneurial intention and indirectly through entrepreneurial self-efficacy. University students are sensitive to entrepreneurial enthusiasm and emotions; however, existing studies rarely take university students as research samples to explore how entrepreneurial intention develops from the perspective of entrepreneurial passion. Thus, this study is one of the first to empirically investigate the mechanism of the EP-EI link by taking university students as potential entrepreneur samples. The findings of this study enrich the research on the antecedents of entrepreneurial intention and reveal the mediating mechanism of how entrepreneurial passion affects entrepreneurial intention.

Third, regarding the effect of entrepreneurial education on individual entrepreneurial intention, the existing studies show mixed results. Some empirical studies show that there is a significant difference between the level of entrepreneurial intention in accepting entrepreneurial education and the students who do not receive entrepreneurship education (Fayolle et al., 2006; Ali, 2013; Heuer and Kolvereid, 2014; Solesvik et al., 2014). However, other studies have found that entrepreneurship education has no or even a negative impact on entrepreneurial intention (Marques et al., 2012). This result indicates that the role of entrepreneurship education in the process of individual entrepreneurial intention must be further explored. The findings of this study enrich the research on effects of entrepreneurial education by exploring the mechanism as to how entrepreneurial education affects entrepreneurial intention. In particular, this study reveals that entrepreneurial self-efficacy plays a fully mediating role between entrepreneurial education and entrepreneurial intention. Thus, entrepreneurial education could enhance entrepreneurial self-efficacy by offering necessary entrepreneurial knowledge and techniques, which could further affect entrepreneurial intention (Ali, 2013).

Lastly, recent studies attach importance to role models in the intention to pursue entrepreneurial activities, however, very few studies have fully uncovered the mechanism of how role models affect entrepreneurial intention (Auken, 2006). In this respect, some extant studies show that role models affect entrepreneurial intention when combined with positive attitudes and entrepreneurial self-efficacy (Nowiński and Haddoud, 2019). Of note, this study has one important and novel finding: role models only have a slight direct impact on entrepreneurial intention but fail to have an indirect effect on entrepreneurial intention through entrepreneurial self-efficacy. The possible reasons could be the role models’ negative signals, lack of direct respondent involvement in entrepreneurial activities and active interaction between role models and respondents, which could contribute to understanding how role models affect entrepreneurial intention and enrich the research on the mechanism behind the RM-EI link.

### Practice Implications

As far as the practical implications of this study are concerned, the findings of this study support a recommendation to enhance the entrepreneurial intention of university students; thus, the specific contexts involved should be considered, as the findings of this study indicate associations between EP-EI, EE-EI, and RM-EI. First, regarding the EP-EI link, the entrepreneurial passion of university students could be stimulated by providing entrepreneurial consulting and financial supporting services, continuously fostering an entrepreneurial atmosphere and culture in the university, and promoting governmental policies that support entrepreneurial activities, all of which could help translate the entrepreneurial passion of university students into entrepreneurial intention and action.

Second, as for the EE-EI link, the design of entrepreneurial education programs should consider the function of not only providing management skills but also enhancing entrepreneurial self-efficacy, as these are interconnected determinants. To enhance ESE, programs and training in entrepreneurial education should ensure university students’ identity as potential entrepreneurs, the feasibility of achieving an entrepreneurial outcome, and the benefits of entrepreneurial activities. Recent research on entrepreneurial education indicates that to enhance and develop entrepreneurial intention, it is recommended that ESE be added to various entrepreneurial programs encompassing design-thinking workshops, pitch-meeting simulations, elevator talks, creativity workshops, and brain-storming (Detienne and Chandler, 2004; Huq and Gilbert, 2017).

Lastly, as for the RM-EI link, the results of this study show that negative interaction between role models and respondents would discourage respondents from developing interest in entrepreneurship. Thus, it is suggested that both respondents’ involvement in entrepreneurial activities and the direct role model–respondent interaction would have a significant impact on respondents’ entrepreneurial self-efficacy and entrepreneurial intention to undertake entrepreneurial action.

In sum, our findings enrich the research on the influencing factors of entrepreneurial intention, provide a theoretical basis for formulating policies to encourage university students’ entrepreneurial intention and help to explore effective ways to enhance entrepreneurial intention and behavior. Future studies should investigate students’ entrepreneurial intention along with their subsequent entrepreneurial behavior (Linan, 2004). This longitudinal research could be used to explore how entrepreneurial intention as shown in the results of this study would be materialized or realized and validate the proposed research model of this study. In-depth interviews regarding why students do or do not pursue entrepreneurial careers could also be conducted.

## Ethics Statement

An ethics approval was not required as per applicable institutional and national guidelines and regulations. The informed consent of the participants was implied through survey completion.

## Author Contributions

FH performed the research design, methodology, literature review, statistical analysis and wrote the manuscript. YS conceived the literature review, research design, and practice implication. ML contributed to the literature review, research design, data collection and assisted in the data analysis. MQ conceived the literature review and practice implication.

## Conflict of Interest Statement

The authors declare that the research was conducted in the absence of any commercial or financial relationships that could be construed as a potential conflict of interest.
